# DISC1 (Disrupted-in-Schizophrenia-1) Regulates Differentiation of Oligodendrocytes

**DOI:** 10.1371/journal.pone.0088506

**Published:** 2014-02-07

**Authors:** Tsuyoshi Hattori, Shoko Shimizu, Yoshihisa Koyama, Hisayo Emoto, Yuji Matsumoto, Natsuko Kumamoto, Kohei Yamada, Hironori Takamura, Shinsuke Matsuzaki, Taiichi Katayama, Masaya Tohyama, Akira Ito

**Affiliations:** 1 Department of Molecular Neuropsychiatry, Graduate School of Medicine, Osaka University, Suita, Osaka, Japan; 2 Department of Anatomy and Neuroscience, Graduate School of Medicine, Osaka University, Suita, Osaka, Japan; 3 Department of Child Development & Molecular Brain Science, United Graduate School of Child Development, Osaka University, Kanazawa University and Hamamatsu University School of Medicine, Suita, Osaka, Japan; 4 Division of Molecular Brain Science, Research Institute of Traditional Asian Medicine, Kinki University, Sayama, Osaka, Japan; 5 Pharmacology Research Laboratories, Dainippon Sumitomo Pharma Co, Ltd, Suita, Osaka, Japan; 6 Department of Neurobiology and Anatomy, Graduate School of Medical Sciences, Nagoya City University, Nagoya, Aichi, Japan; Universidade Federal do ABC, Brazil

## Abstract

*Disrupted-in-schizophrenia 1 (DISC1)* is a gene disrupted by a translocation, t(1;11) (q42.1;q14.3), that segregates with major psychiatric disorders, including schizophrenia, recurrent major depression and bipolar affective disorder, in a Scottish family. Here we report that mammalian DISC1 endogenously expressed in oligodendroglial lineage cells negatively regulates differentiation of oligodendrocyte precursor cells into oligodendrocytes. DISC1 expression was detected in oligodendrocytes of the mouse corpus callosum at P14 and P70. DISC1 mRNA was expressed in primary cultured rat cortical oligodendrocyte precursor cells and decreased when oligodendrocyte precursor cells were induced to differentiate by PDGF deprivation. Immunocytochemical analysis showed that overexpressed DISC1 was localized in the cell bodies and processes of oligodendrocyte precursor cells and oligodendrocytes. We show that expression of the myelin related markers, CNPase and MBP, as well as the number of cells with a matured oligodendrocyte morphology, were decreased following full length DISC1 overexpression. Conversely, both expression of CNPase and the number of oligodendrocytes with a mature morphology were increased following knockdown of endogenous DISC1 by RNA interference. Overexpression of a truncated form of DISC1 also resulted in an increase in expression of myelin related proteins and the number of mature oligodendrocytes, potentially acting via a dominant negative mechanism. We also identified involvement of Sox10 and Nkx2.2 in the DISC1 regulatory pathway of oligodendrocyte differentiation, both well-known transcription factors involved in the regulation of myelin genes.

## Introduction

DISC1 gene is specifically disrupted by a t(1;11) (q42.1;q14.3) balanced translocation, in a large Scottish pedigree, which leads to several major mental illnesses, such as schizophrenia (SZ), bipolar affective disorder and recurrent major depression [1]–[3]. Many subsequent genetic studies indicated that DISC1 is not only implicated in schizophrenia and mood disorders, but also in autism spectrum disorders, Asperger syndrome, attention deficit and hyperactivity disorder (ADHD) and agenesis of the corpus callosum [4]–[9]. Biological studies have shown that DISC1 plays a role in multiple process of brain development such as neuronal proliferation, migration, differentiation, and modulation of *DISC1* gene in rodents causes behavioral changes [10]–[21].

Multiple lines of evidence, obtained by brain imaging, studies in postmortem brains and genetic association studies, have implicated oligodendrocytes and myelin dysfunction in SZ, major depressive disorder (MDD), autism and ADHD [22]–[24]. Specifically, compromised white matter (WM)/myelin integrity, a reduced number and/or altered morphology of oligodendrocytes, and the aberrant expression and genetic association of oligodendrocytes/myelin-related genes have been identified by a number of studies [25]–[32]. It can also be inferred from the higher than chance co-occurrence of WM-diseases, such as multiple sclerosis (MS), leukodystrophies and velocardiofacial syndrome, with SZ-like psychoses [33]–[35], that oligodendrocytes and myelin dysfunction may play a key role in the pathophysiology of mental illness.

Despite substantial evidence indicating the role of oligodendrocyte abnormalities in pathophysiology of psychosis, neurobiological studies have predominantly focused on neurons. Accordingly, a large number of studies have shown the key role of DISC1 in neurons [10]–[19], while only a handful of studies have addressed a possible role of DISC1 in oligodendrocytes [36]–[38]. DISC1 expression in human brain and primary cultured rat cortical oligodendrocytes was shown by Seshadri et al. [37] and a critical requirement for DISC1 in oligodendroglial development, by promoting specification of olig2-positive cells in the hindbrain and other brain regions of zebrafish, was reported by Wood *et al* [36]. Nevertheless, no study to date, has directly addressed the functional role of DISC1 expressed in a mammalian cell of glial lineage.

By examining the effect of RNA interference (RNAi) on endogenous DISC1, and also overexpression of either truncated DISC1 or full length DISC1, we here show for the first time that endogenous DISC1 expressed in glial cells negatively regulates mammalian oligodendrocyte development *in vitro*, acting upstream of Sox10 and/or Nkx2.2.

## Materials and Methods

### Ethics Statement

The study protocol was approved by the Institutional Animal Care and Use Committee of Osaka University (No. 20-138-006).

### Antibodies

Antibodies used in this study are as follows: anti-CNPase (Sigma, St. Louis, MO, USA); anti-APC (Millipore, Billerica, MA, USA); anti-MBP (Millipore); anti-β-tubulin (Sigma); anti-GFP (Abcam, Cambridge, MA, USA); anti-GAPDH (Santa Cruz Biotechnology, Santa Cruz, CA, USA); HRP-linked anti-IgG (Cell Signaling, Danvers, MA, USA); Alexa-Flour568-anti-IgG and Alexa-Flour488-anti-IgG (Molecular Probes). DAPI was purchased from Invitrogen.

### Plasmids and Adenovirus

Complementary DNAs coding full length human DISC1 and truncated human DISC1 (1–598) were cloned into pEGFP-C1. A recombinant adenovirus expressing either GFP, human DISC1 with GFP fused to the C-terminus, or truncated human DISC1 (1–598) with GFP fused to the C-terminus (GFP-Adv, DISC1-GFP-Adv and trDISC1-GFP-Adv, respectively) was generated using the ViraPower Adenoviral Expression System (Invitrogen), according to the manufacturer’s instructions.

### *In situ* Hybridization-immunohistochemistry

cDNA fragments of mouse DISC1 were amplified by reverse-transcribed-PCR using the sense/antisense primer set of 5′- ATGCAGGGCGGGGGTCCCCGG -3′/5′-TCAGGCCTCGGTTTCCTGAG-3′, and used as templates for probe synthesis. Probe was hydrolyzed and *in situ* hybridization of coronal mouse brain sections with DIG-labeled RNA probes was performed as described previously [39]. The slides were washed thoroughly in PBS following a colorimetric reaction. Next, the slides were incubated overnight at 4°C with the primary antibody (monoclonal mouse anti-APC antibody) at 1∶50 in PBS. After washing in PBS, the slides were incubated for 30 min at RT with the secondary antibody (biotinylated anti-mouse IgG antibody from Vector Laboratories). After amplification with the avidin-biotin complex using ABC kit (Vector Laboratories), reaction products were visualized with 50 mM Tris-HCl buffer (pH 7.6) containing 0.02% diaminobenzidine tetrahydrochloride (Sigma) and 0.01% hydrogen peroxide. After dehydration, the sections were sealed using Entellan.

### siRNA Design and RNA Interference

The targeted sequences of rat DISC1 were: 5′-GGCTACATGAGAAGCACAG-3′ (DISC1-siRNA-1) and 5′-CTGGCTGATGCGAGAGAAA-3′ (DISC1-siRNA-2). The DISC1-siRNA-1 was previously shown to knockdown mouse and rat DISC1 [15], [40], [41]. The DISC1-siRNA-2 was designed using online software tool siDirect. We validated knockdown of endogenous rat DISC1 transcripts by transfecting DISC1-siRNA-1 or DISC1-siRNA-2 in oligodendrocyte precursor cells. The targeted sequences of rat Sox10 and Nkx2.2 siRNAs were: 5′-CTGTGTCACTGTCCTAAA-3′ (Sox10-siRNA) and 5′-GTTTGTGTGAGTAGCGATA-3′ (Nkx2.2-siRNA). A scrambled sequence (5′-GCGCGCTTTGTAGGATTCGT-3′) was used as a control. Transfection of these siRNAs was carried out using RNAiMAX (Invitrogen), according to the manufacturer’s protocol. To assess the transfection efficiency of siRNA, cells were transfected with Block-iT Alexa Fluor Red Fluorescent Oligo (Invitrogen) and examined under fluorescence microscope 24 hours later.

### *In vitro* Oligodendrocyte Differentiation and Transfection

Primary cultures of rat oligodendrocyte precursor cells were established and induced to differentiate into oligodendrocytes according to the method of Chen *et al*., with some modification [42]. Single cell suspensions of P1 rat cortex was prepared in MEM supplemented with 0.292 g/l L-glutamine, 4 g/l D-glucose, 3.2 g/l NaHCO_3_ and 10% FBS, and plated on poly-L-lysine (PLL) coated culture flasks (Nunc). These mixed brain cell cultures were cultured in humidified CO_2_ incubators for 10 to 14 days with the medium changed every 3 days. Twelve hours after the last medium change, the flask was rotated at 200 rpm for 20 hours to dislodge glial lineage cells. Dislodged cells were plated on non-coated dishes and incubated for 1 hour to allow astrocytes and microglia to adhere to the dish. Oligodendrocyte precursor cells were collected as non-adherent cells, re-suspended in proliferation medium (PM: Neurobasal medium (Invitrogen) supplemented with 5 ng/ml insulin (Sigma), 5 ng/ml NT3 (Pepro Tech Inc., Rocky Hill, NJ, USA), 10 ng/ml PDGF (Wako, Osaka, Japan), 2 mM L-glutamine (Sigma) and B27 (Invitrogen)). Oligodendrocyte precursor cells plated on PLL coated flasks were maintained for 3 days with half-medium-changes with PM every second day. Differentiation of oligodendrocyte precursor cells to oligodendrocytes was induced by replacing the whole medium with PM deprived of PDGF (0 hours). After the induction of differentiation, cells were maintained with half-medium-changes with PDGF deprived PM. Cells were infected with adenovirus expressing GFP, DISC1-GFP or trDISC1-GFP, 12 hours before PDGF deprivation at 0 hours. We confirmed that proportion of cells positive for oligodendrocyte precursor cells marker was 91.8±2.4% of the whole cell population at 0 hours by immunostaining with anti-NG2 antibody. Transfection of siRNAs was performed at 0 hours and the whole medium was changed with PM 4 hours after transfection. In rescue experiments, cells were infected with adenovirus expressing GFP or DISC1-GFP 24 hours after the siRNA transfection and the whole medium was changed with PM 12 hours after the infection.

### Quantitative Real-time PCR (qRT-PCR)

Total RNA was reverse-transcribed using High-Capacity cDNA Reverse Transcription Kits (Applied Biosystems, Warrington, UK), and analyzed by RT-PCR to determine expression levels of *DISC1*, *CNPase*, *MBP*, *Sox10*, *Nkx2.2,* β*-actin* and *GAPDH*. For *DISC1* gene, two sets of forward/reverse primers were used: *DISC1* 5′- TGGCTGTCCCTAGAACACCC-3′/5′- CTCATGCCTATGGCTTCGC-3′ (DISC1 primer-1) or 5′-TGATGCGAGAGAAAGAGCAA-3′/5′- AGCATCTCCTGATCCTCCAA-3′ (DISC1 primer-2). DISC1 primer-1 and DISC1 primer-2 target to exon 11 to 12 and exon 5 to 6 of rat *DISC1* gene, respectively. The following sets of forward/reverse primers were used for other genes: *CNPase* 5′-CAACAGGATGTGGTGAGGA-3′/5′-CTGTCTTGGGTGTCACAAAG-3′, *MBP* 5′-CACACACAAGAACTACCCA-3′/5′-CACACACAAGAACTACCCA-3′, *Sox10* 5′-AGCCCAGGTGAAGACAGAGA-3′/5′-CCCCTCTAAGGTCGGGATAG-3′ and *Nkx2.2* 5′-CGGGCTGAGAAAGGTATGGA-3′/5′-TGTGCTGTCGGGTACTGGG-3′. To standardize the experiments, we designed primer sets (5′-GCCTTCTCTTGTGACAAAGTGG-3′/5′-ATTCTCAGCCTTGACTGTGCC-3′) and (5′-CCTGTATGCCTCTGGTCGTA-3′/5′-CCATCTCTTGCTCGAAGTCT-3′) to amplify a portion of the rat *GAPDH* and β*-actin* gene respectively. RT-PCR was set up using Power SYBR Green PCR Master Mix (Applied Biosystems). Comparison of specific ratios (gene of interest/*GAPDH or* β*-actin*) was used to assess differences in expression levels between groups.

### Western Blotting

Cells were lysed with lysis buffer (50 mM Tris-HCl, pH 7.4, containing 50 mM NaCl, 1 mM EDTA, 1% Triton X-100, 0.1% SDS, 0.5% sodium deoxycholate and protease inhibitor mixture). Western blotting was performed as described previously [43].

### Immunocytochemical Analysis

Immunocytochemistry was performed as described [44]. Mouse anti-β-tubulin, rabbit anti-GFP and mouse anti-CNPase antibodies were all used at dilutions of 1∶200. Confocal microscopy was performed using an LSM-510 laser scanning microscope (Carl Zeiss, Oberkochen, Germany). For the analysis of morphological differentiation, cells were classified to one of the following morphological categories: simple, bipolar or stellate cells having short primary branches; intermediate, cells having very long primary branches and/or secondary branches; or complex morphology, cells with tertiary branches [45].

### Statistical Analyses

Unless otherwise mentioned, all statistical comparisons were determined by Student’s *t*-test, with significant differences indicated by *p*<0.05.

## Results

### DISC1 is Expressed in Oligodendrocytes in the Mouse Corpus Callosum

**Figure 1:**
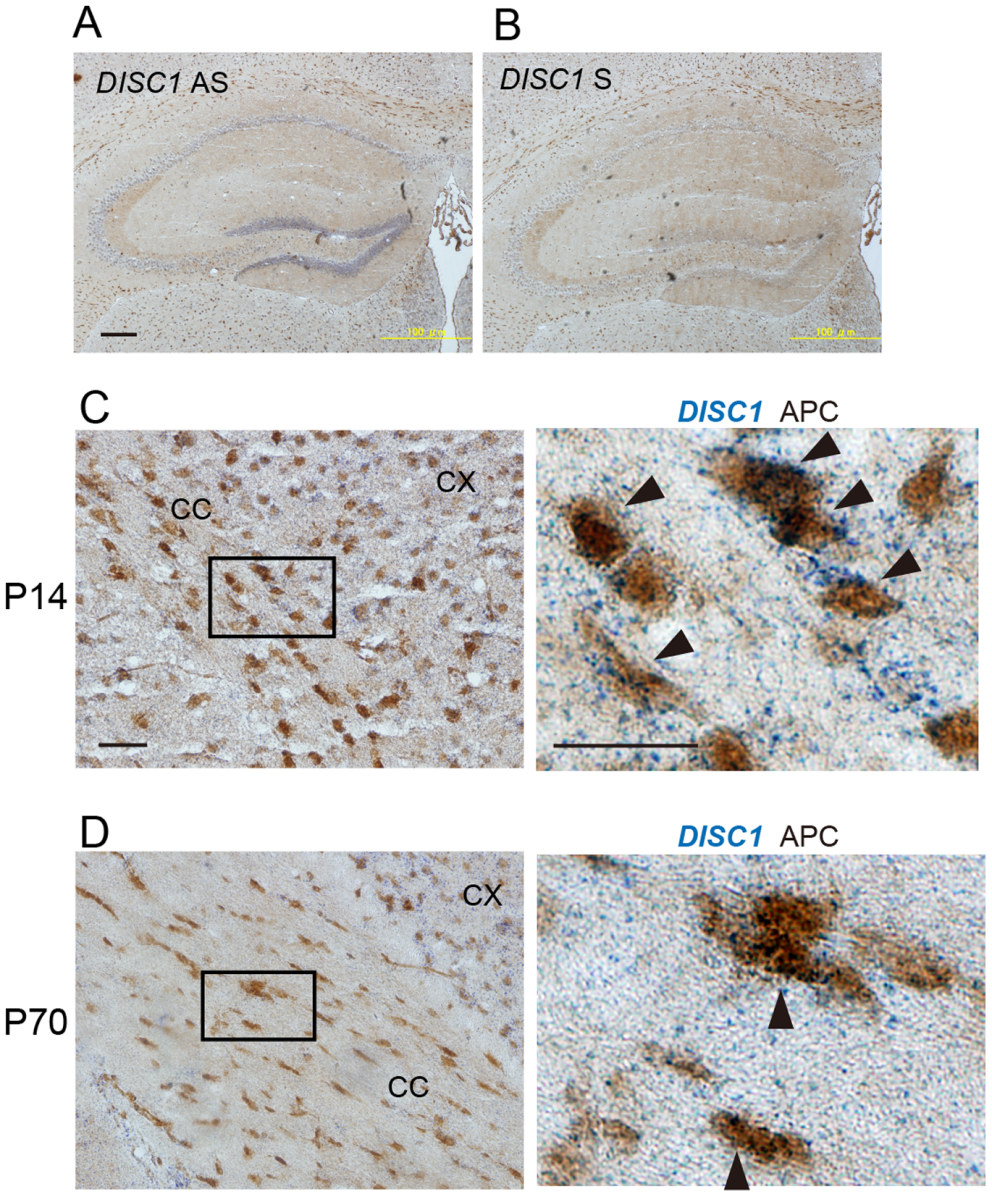
DISC1 mRNA is expressed in oligodendrocytes in the corpus callosum of mouse brain. **A** Double *In situ* hybridization-immunohistochemistry analysis of hippocampal sections from P70 mice with the antisense RNA probe to DISC1 and antibody against APC. Scale bar, 200 µm. H: hippocampus AS: antisense **B** As controls, adjacent sections were hybridized with DIG-labeled sense RNA probe. S: sense **C, D,** Double *In situ* hybridization-immunohistochemistry analysis of brain sections from P14 and P70 mice demonstrates the expression of DISC1 mRNA in APC expressing oligodendrocytes in the corpus callosum of mouse at P14 (**C**) and P70 (**D**). High magnified images of the squared region in the left panels are shown in the adjacent right panels. Arrowheads indicate DISC1+/APC+ cells. Scale bars, 50 µm.

Although a previous study reported DISC1 expression in oligodendrocytes in human brain, DISC1 expression in oligodendroglial lineage cells in mouse brain had not been investigated [37]. To investigate whether DISC1 transcripts are expressed in oligodendrocytes of mouse brain, we performed *in situ* hybridization-immunohistochemistry using DIG-labeled RNA probe for mouse *DISC1* and anti-APC antibody. Consistent with previous studies [41], [46], DISC1 mRNA was highly expressed in the hippocampus at P70 (Fig. 1 A). No signal was detected in sections hybridized with the sense probe confirming the specificity of our *in situ* hybridization probe (Fig. 1 B). DISC1 mRNA was found in cells expressing APC, an oligodendrocyte marker, both at P14 (Fig. 1 C) and P70 (Fig. 1 D). Furthermore, higher expression of DISC1 mRNA in oligodendrocytes at P14 than P70 was suggested. These results show that DISC1 is expressed in oligodendrocytes of mouse brain.

### DISC1 Expression Decreases in the Course of *in vitro* Oligodendrocyte Differentiation

**Figure 2:**
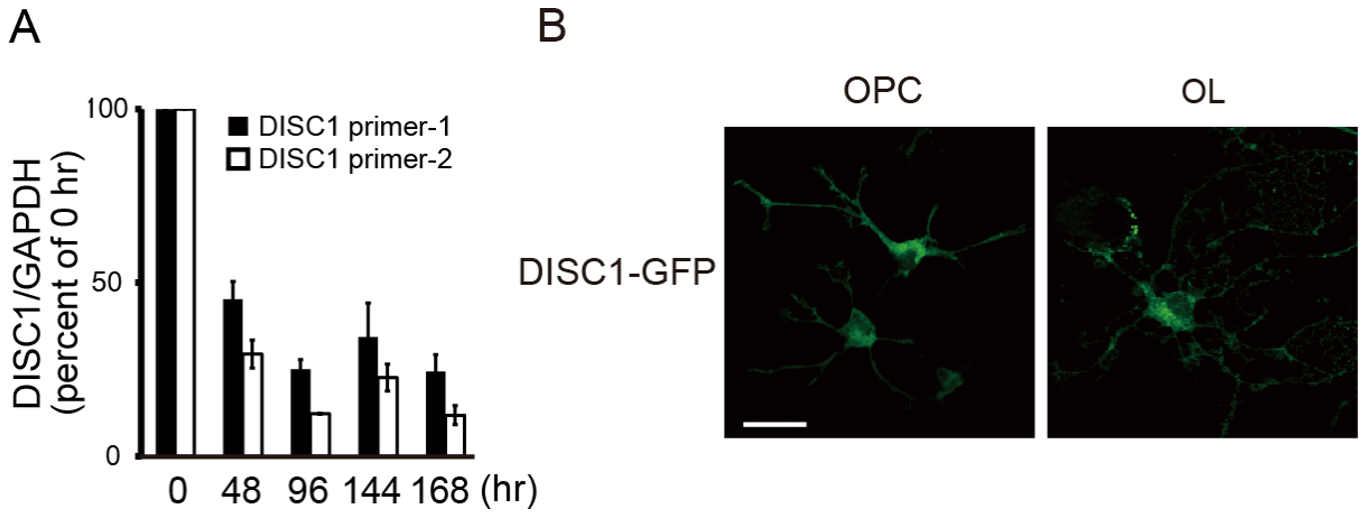
DISC1 expression decreases in the course of oligodendrocyte differentiation. **A**, Primary cultured cells were harvested at indicated times after PDGF deprivation and mRNA was quantified by qRT-PCR. Data are expressed as the mean±s.e.m. of at least three independent experiments. DISC1 mRNA level at 0 hr is higher than later time-points with p<0.01 by one-way ANOVA followed by Tukey’s test. **B,** Intracellular localization of overexpressed DISC1-GFP in cultured oligodendrocyte precursor cells and oligodendrocytes. Cells were immunostained with anti-GFP antibody. Scale bar = 50 µm.

As a developmental decrease of DISC1 mRNA in the mouse corpus callosum was suggested, we investigated DISC1 expression during *in vitro* differentiation of oligodendrocyte precursor cells to oligodendrocytes. Primary cultured rat oligodendrocyte precursor cells were induced to differentiate to oligodendrocytes by depriving PDGF from the culture medium. Quantitative PCR analysis using two sets of primers for *DISC1* showed that DISC1 mRNA expression was reduced after PDGF deprivation (Fig. 2 A). The decrease of DISC1 expression was confirmed using DISC1 primer-1 and another reference gene (β-actin) (100% for 0 h; 48.9±11.3% for 48 h; 26.0±4.8% for 96 h; 36.4±13.5% for 120 h; 22.6±3.8% for 144 h). These results suggest that DISC1 is involved in differentiation of oligodendrocyte lineage cells. Next, we examined the subcellular localization of overexpressed DISC1 in primary cultured oligodendrocyte precursor cells and oligodendrocytes by immunocytochemistry. Overexpressed DISC1 was preferentially expressed in the cell soma and cytoplasmic processes of oligodendrocytes, with marginal nuclear expression. In oligodendrocyte precursor cells, subcellular localization of DISC1 was similar to oligodendrocytes (Fig. 2 B). These results demonstrate that DISC1 expression is more abundant in oligodendrocyte precursor cells and DISC1 protein is expressed in cell soma and processes in oligodendrocyte precursor cells and oligodendrocytes.

### DISC1 Overexpression Retards Oligodendrocyte Differentiation

**Figure 3:**
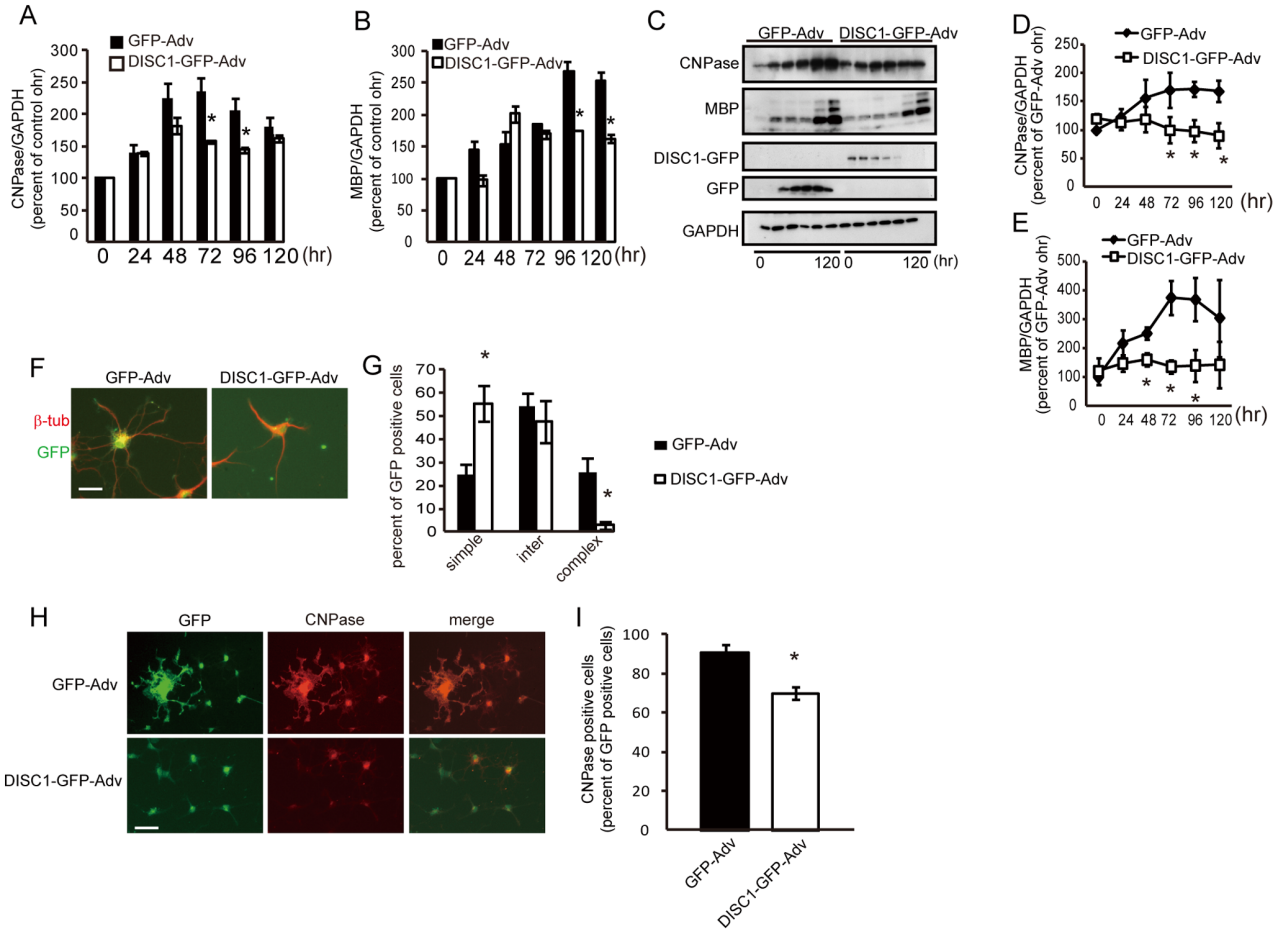
DISC1 overexpression inhibits oligodendrocyte differentiation. **A, B,** Cells infected with GFP-Adv or DISC1-GFP-Adv were harvested at indicated times after PDGF deprivation and mRNA levels of CNPase (**A**) and MBP (**B**) were quantified by qRT-PCR. Data are expressed as mean ± s.e.m. of at least three independent experiments. *p<0.05 vs. GFP-Adv. **C**, Cells infected with GFP-Adv or DISC1-GFP-Adv were lysed at 0, 24, 48, 72, 96 and 120 hours after PDGF deprivation and subjected to western blot analysis. **D, E,** Quantitation of relative band densities for CNPase (**D**) and MBP (**E**) were performed by scanning densitometry. Data are expressed as mean ± s.e.m. of at least three independent experiments. *p<0.05 vs. GFP-Adv. **F–I,** Oligodendrocyte precursor cells were infected with GFP-Adv or DISC1-GFP-Adv for 12 hours and induced to differentiate by PDGF deprivation for 96 hours then fixed for immunostaining. **F, G,** Cells were immunostained with anti-GFP and anti-β-tubulin antibodies for morphological observation. Infected cells from three independent cultures were classified according to their morphology (simple, intermediate, or complex) and quantified. The percentage of cells within each category, relative to the total number of GFP positive cells, is shown. *p<0.05 vs. GFP-Adv. Scale bar = 50 µm. **H, I,** Cells were immunostained with anti-GFP and anti-CNPase antibodies. The percentage of CNPase positive cells relative to the total number of GFP positive cells is shown. Infected cells from three experiments were analyzed. *p<0.05 vs. GFP-Adv. Scale bar = 100 µm.

To explore the functional role of decreased expression of DISC1 in the course of oligodendrocytes differentiation (Fig. 2A), DISC1 was overexpressed in oligodendrocyte precursor cells using a DISC1 expressing adenovirus (DISC1-GFP-Adv). In cells induced to differentiate 12 hours after DISC1-GFP-Adv infection, expression of the myelin genes, CNPase (Fig. 3 A, C, D) and MBP (Fig. 3 B, C, E), were decreased at both the mRNA and protein level, compared with control (GFP-Adv) infected cells. To confirm the reduced CNPase expression in DISC1 overexpressing cells, we determined the ratio of GFP expressing cells with CNPase expression in GFP-Adv or DISC1-GFP-Adv infected cells by immunostaining with anti-CNPase antibody. The percentage of CNPase immuno-positive cells decreased 96 hours after PDGF deprivation, following infection of DISC1-GFP-Adv (70.1±3.3%) compared with GFP-Adv (90.6±2.5%) (Fig. 3 H, I).

Morphological transformation of cells infected by GFP-Adv or DISC1-GFP-Adv was examined 96 hours after PDGF deprivation. Infected cells were identified by GFP expression and their morphology was assessed by immunostaining with anti-β-tubulin. The morphology of infected cells was classified as simple, intermediate or complex, as described previously [45]. Of 238 GFP-Adv infected cells, 24.9±3.4% showed a complex morphology, characterized by the presence of several interlaced fine branches indicative of advanced differentiation. Undifferentiated cells, showing a simple morphology defined by primary branches and the absence of secondary and tertiary processes, comprised 23.6±2.2% of the total. In contrast, of 191 DISC1-overexpressing cells, only 2.5±0.8% displayed a complex morphology and 53.3±3.9% showed a simple morphology (Fig. 3 F, G). Thus, DISC1 overexpression results in a reduction of cells with a complex morphology, and an increase in cells with a simple morphology, suggesting that decrease in endogenous DISC1 expression upon PDGF deprivation in oligodendrocyte precursor cells has a functional role to promote oligodendrocyte differentiation.

### DISC1 Knockdown Promotes Oligodendrocyte Differentiation

**Figure 4:**
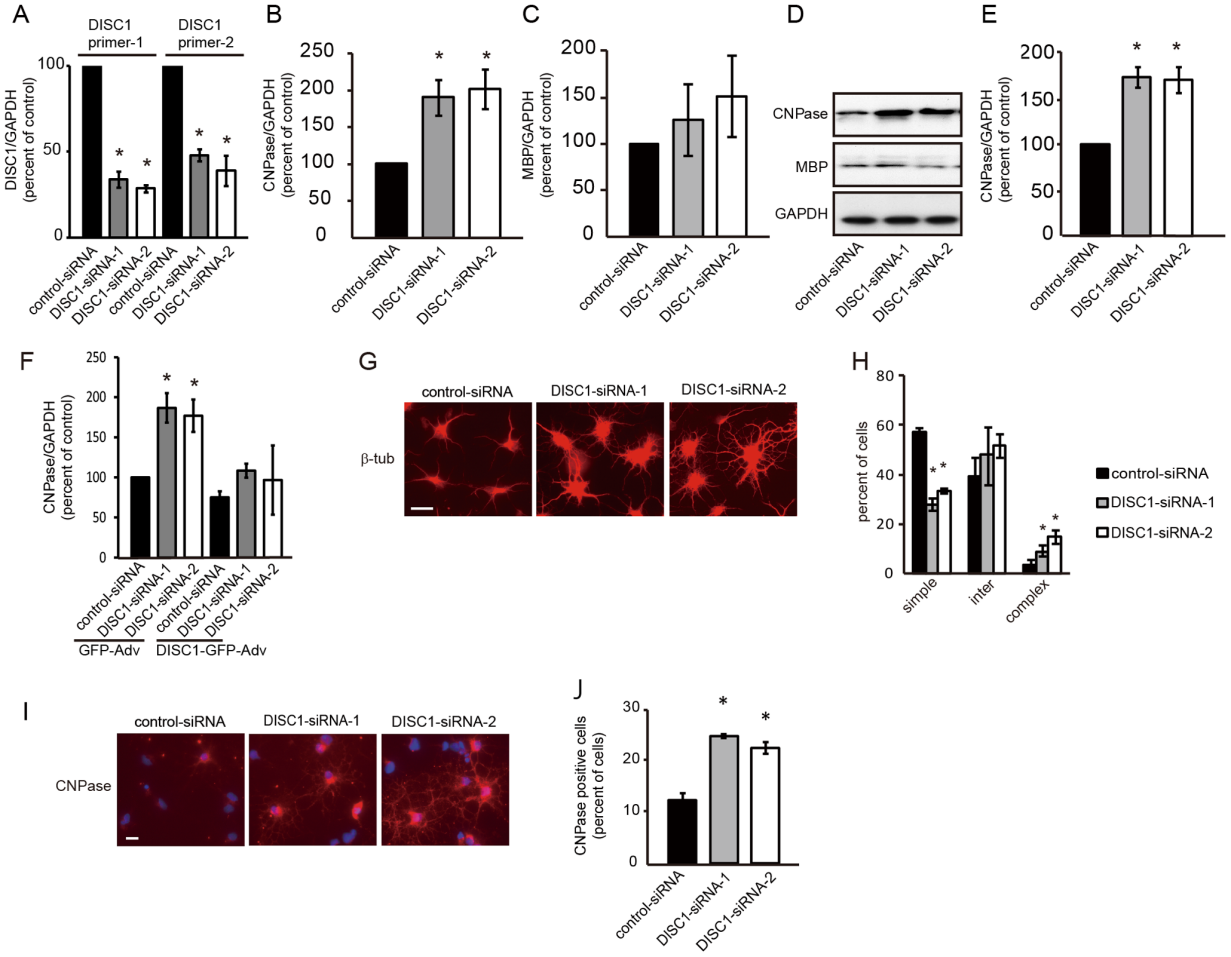
DISC1 knockdown promotes oligodendrocyte differentiation. **A–C**, Cells transfected with control-siRNA, DISC1-siRNA-1 or DISC1-siRNA-2 were cultured for 24 (**A**)or 48 (**B,C**) hours in medium containing PDGF and mRNA levels of DISC1 (**A**), CNPase (**B**) and MBP (**C**) were quantified by qRT-PCR. Data are expressed as mean ± s.e.m. of at least three independent experiments. *p<0.05 vs. control-siRNA. **D,** Cells transfected with control-siRNA, DISC1-siRNA-1 or DISC1-siRNA-2 were lysed 72 hours after siRNA transfection and analyzed by western blotting. **E**, Quantitation of CNPase was performed by scanning densitometry. Data are expressed as mean ± s.e.m. of at least three independent experiments. *p<0.05 vs. control-siRNA. F, DISC1 knockdown mediated increase of CNPase mRNA was rescued by overexpression of DISC1. Cells were infected with GFP- or DISC1-GFP-Adv 24 hours after control- or DISC1-siRNA transfection. Forty-eight hours after the infection, mRNA level of CNPase was quantified by qRT-PCR. Data are expressed as mean ± s.e.m. of at least three independent experiments. *p<0.05 vs. control-siRNA and GFP-Adv. **G–J** Oligodendrocyte precursor cells were transfected with control-siRNA, DISC1-siRNA-1 or DISC1-siRNA-2 and cultured in medium containing PDGF for 72 hours then fixed for immunostaining. Cells were immunostained with anti-β-tubulin antibody (**G, H**) or anti-CNPase-antibody (**I, J**) and analyzed as described in figure legend 3. *p<0.05 vs. control-siRNA. Scale bars = 50 µm.

To further investigate the role of endogenous DISC1 in oligodendrocyte differentiation, we treated oligodendrocyte precursor cells with DISC1 specific siRNA (DISC1-siRNA) and examined mRNA or protein expression levels of CNPase and MBP 48 or 72 hours after siRNA transfection. To isolate the effect of DISC1 knockdown induced by DISC1-siRNA, cells were maintained in medium with PDGF during the course of the experiment. The proportion of siRNA-transfected oligodendrocyte precursor cells determined using Block-iT Alexa Fluor Red Fluorescent Oligo was 93.6±1.2% of total cell population. Two DISC1-siRNAs (DISC1-siRNA-1 and DISC1-siRNA-2) targeting exon2 and exon5 of the DISC1 gene respectively have already been shown to effectively suppress rat DISC1 protein expression [15], [40], [41]. Suppression of DISC1 expression by these siRNAs was confirmed by qRT-PCR with two different primer sets for rat DISC1 (DISC1 primer-1 (100% for control siRNA; 33.7±4.8% for DISC1-siRNA-1; 28.5±2.1% for DISC1-siRNA-2); DISC1 primer-2 (100% for control siRNA; 47.9±3.6% for DISC1 siRNA-1; 39.0±8.8% for DISC1 siRNA-2)) (Fig. 4 A). Effective knockdown of DISC1 expression was also confirmed using another reference gene (β-actin) (data not shown). Transfection of either of two siRNAs for DISC1 resulted in an increase of CNPase, at both the mRNA and protein level, compared with control-siRNA treated cells (Fig. 4 B, D, E). Although not statistically significant, we also observed a trend towards increased expression of MBP (Fig. 4 C, D). Immunostaining of *in vitro* oligodendroglial lineage cells with anti-CNPase antibody revealed that DISC1-siRNA treatment increased the percentage of CNPase positive cells (DISC1-siRNA-1: 24.6±0.7%; DISC1-siRNA-2: 22.3±1.9%), compared with control-siRNA treated cells (12.2±2.3%) (Fig. 4 I, J). To confirm the effect of DISC1 knockdown on expression of CNPase, we performed rescue experiment by co-expression of DISC1 siRNAs and human DISC1-GFP. Human DISC1 rescued the increased CNPase expression by DISC1 siRNA (Fig. 4 F). Morphological transformation of cells transfected with control-siRNA or DISC1-siRNAs was examined by immunostaining for β-tubulin 72 hours after siRNA transfection. Of 225 control-siRNA transfected cells, 57.4±0.9% showed a simple morphology and only 3.5±0.7% of cells displayed a complex morphology. In contrast, DISC1 knockdown decreased the cells with a simple morphology (DISC1-siRNA-1: 27.9±4.6%; DISC1-siRNA-2: 33.5±1.3%), and increased cells with a complex morphology (DISC1-siRNA-1: 6.2±2.7%; DISC1-siRNA-2: 14.7±1.7%) (Fig. 4 G, H). Our results suggest that endogenous DISC1 in an oligodendrocyte lineage cell negatively regulates oligodendrocyte differentiation.

### Overexpressed Truncated-DISC1 Promotes Oligodendrocyte Differentiation

**Figure 5:**
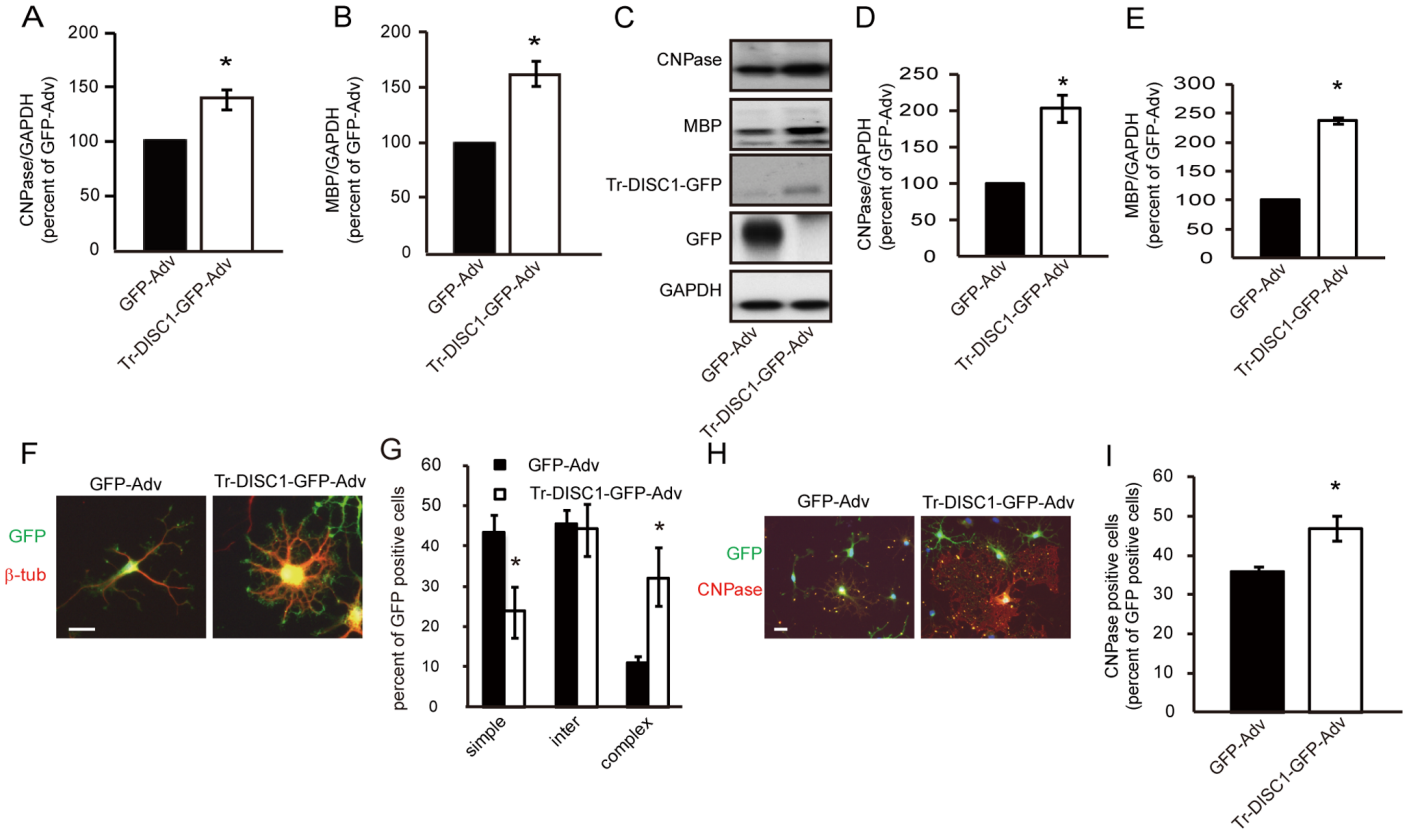
Overexpressed truncated DISC1 promotes oligodendrocyte differentiation. **A–E** Effect of truncated DISC1 overexpression on CNPase and MBP expression. Oligodendrocyte precursor cells were infected with GFP-Adv or trDISC1-GFP-Adv for 12 hours and induced to differentiate by PDGF deprivation for 36 hours. mRNA levels of CNPase (**A**) and MBP (**B**) were quantified by qRT-PCR. Data are expressed as mean ± s.e.m. of at least three independent experiments. *p<0.05 vs. GFP-Adv. **C,** Cells infected with GFP-Adv or trDISC1-GFP-Adv for 12 hours were lysed 60 hours after PDGF deprivation and subjected to western blot analysis. **D, E,** Quantitation of relative band densities for CNPase (**D**) and MBP (**E**) was performed by scanning densitometry. Data are expressed as mean ± s.e.m. of at least three independent experiments. *p<0.05 vs. GFP-Adv. **F–I** Oligodendrocyte precursor cells were infected with GFP-Adv or trDISC1-GFP-Adv for 12 hours and induced to differentiate by depriving PDGF for 60 hours then fixed for immunostaining. Cells were immunostained for GFP and β-tubulin (**F, G**) or for GFP and CNPase (**H, I**) and analyzed as described in figure legend 3. *p<0.05 vs. GFP-Adv. Scale bar = 50 µm.

To further confirm the negative regulatory role of DISC1 in oligodendrocyte differentiation, we overexpressed truncated DISC1, which has been suggested to be generated when the DISC1 gene is disrupted by the balanced translocation of chromosome 1. Truncated DISC1 is predicted to function in a dominant negative fashion, possibly by competing with the full-length form for interacting proteins [12]. When oligodendrocyte precursor cells infected with a truncated DISC1 expressing adenovirus (trDISC1-GFP-Adv) for 12 hours, were deprived of PDGF, levels of CNPase and MBP, both mRNA and protein levels, were significantly increased compared with control (GFP-Adv) adenovirus infected cells (Fig. 5 A–E). Furthermore, immunostaining analysis with anti-CNPase antibody showed a higher proportion of CNPase positive cells in trDISC1-GFP-Adv, compared with control adenovirus, infected cells (GFP-Adv: 36.0±1.2%; tr-DISC1-GFP-Adv: 46.9±3.1%) (Fig. 5 H, I).

Immunostaining of trDISC1-GFP-Adv infected cells with anti-β-tubulin antibody showed that upon differentiation, there was a higher ratio of cells with a complex morphology compared with control adenovirus infected cells (GFP-Adv: 11.0±0.8%; trDISC1-GFP-Adv: 32.0±4.9%) (Fig. 5 F, G). Conversely, the ratio of cells displaying a simple morphology in trDISC1-GFP-Adv, compared to GFP-Adv, infected cultures was significantly lower (GFP-Adv: 43.4±2.1%; trDISC1-GFP-Adv: 23.9±4.2%). These results suggest that truncated DISC1 expression promotes differentiation of oligodendroglial lineage cells, and provides additional supporting evidence for negative regulation of oligodendrocyte differentiation by endogenous DISC1.

### Involvement of Sox10 and/or Nkx2.2 in the Regulatory Pathway of Oligodendrocyte Differentiation by DISC1

Transcription factors involved in oligodendrocyte specification and differentiation include, but are not limited to, the basic helix-loop-helix (bHLH) family members Olig1 and Olig2, the inhibitor of DNA binding (Id) family of proteins Id2 and Id4, SRY box containing (Sox) family members Sox10, and the homeobox containing (Hox) transcription factor Nkx2.2 [47], [48].

**Figure 6:**
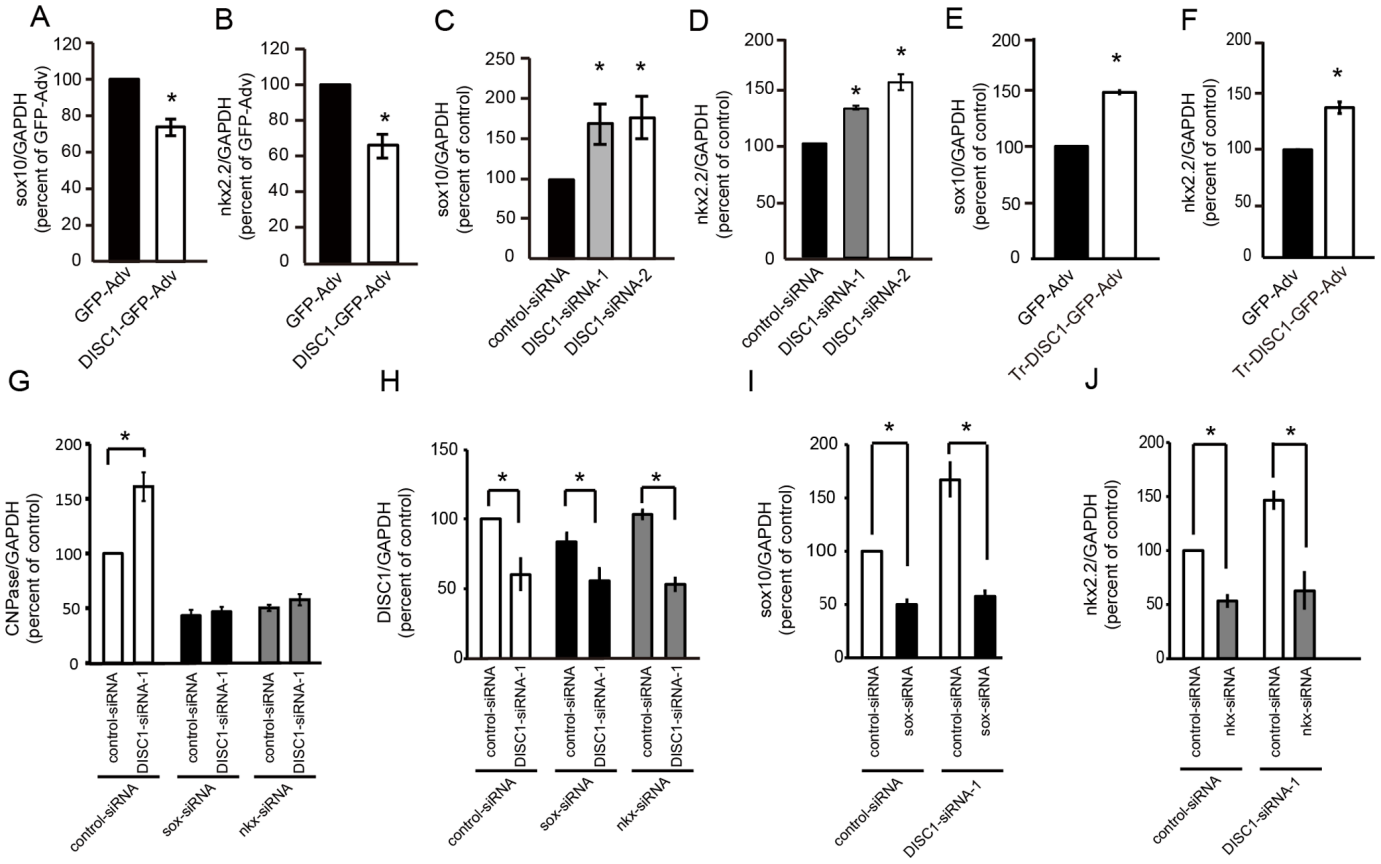
Involvement of Sox10 and/or Nkx2.2 in the regulatory pathway of oligodendrocyte differentiation by DISC1. **A, B,** Expression of Sox10 and Nkx2.2 mRNA were decreased by DISC1 overexpression. Oligodendrocyte precursor cells were infected with GFP-Adv or DISC1-GFP-Adv for 12 hours and induced to differentiate by depriving PDGF for 36 hours. **C, D,** Expression of Sox10 and Nkx2.2 mRNA were increased by DISC1 knockdown. Oligodendrocyte precursor cells were transfected with control-siRNA, DISC1-siRNA-1 or DISC1-siRNA-2 and cultured in medium containing PDGF for 48 hours. **E, F,** Expression of Sox10 and Nkx2.2 mRNA were increased by truncated DISC1 overexpression. Oligodendrocyte precursor cells were infected with GFP-Adv or trDISC1-GFP-Adv for 12 hours and induced to differentiate by PDGF deprivation for 36 hours. **G–J** DISC1 knockdown mediated increase of CNPase mRNA was inhibited by a simultaneous knockdown of either Sox10 or Nkx2.2. Oligodendrocyte precursor cells were co-transfected with control-siRNA or DISC1-siRNA-1 and sox10-siRNA or nkx2.2-siRNA and cultured in medium containing PDGF for 24 (**H**) or 48 hours (**G, I, J**). mRNA quantification was performed 48 hours after adenovirus infection (**A, B, E, F**) or siRNA transfection (**C, D, G, I, J**) or 24 h hours after siRNA transfection (**H**) by qRT-PCR. Data are expressed as mean ± s.e.m. of at least three independent experiments. *p<0.05 vs. GFP-Adv (*A, B*), *p<0.05 vs. control-siRNA (*C, D*), *p<0.05 (*E–H*).

To examine the potential involvement of transcription factors in the regulatory pathway of oligodendroglial lineage cells by DISC1, we investigated the effects of manipulating DISC1 expression on the mRNA expression of transcription factors. Of the transcription factors examined (sox10, nkx2.2, mash1, olig1, olig2, Id2 and Id4), mRNA expression of Sox10 and Nkx2.2 were significantly decreased in DISC1 overexpressing cells (Fig. 6 A, B) while expression level of other transcription factors were not significantly changed compared with control cells (mash1; 104±0.3%, olig1; 107±0.2%, olig2; 99.5±1.6%, Id2; 111.8±6.0%; Id4; 112.0±8.2%). In contrast, knockdown of endogenous DISC1 resulted in enhanced expression of Sox10 and Nkx2.2 (Fig. 6 C, D). Furthermore, truncated form of DISC1 also increased Sox10 and Nkx2.2 expression (Fig. 6 E, F).

We examined CNPase expression levels in cells co-transfected with DISC1-siRNA-1 and siRNAs targeting either Sox10 or Nkx2.2 (sox-siRNA or nkx-siRNA) to elucidate if DISC1 is acting upstream or downstream of these transcription factors. When either Sox10 or Nkx2.2 was simultaneously knocked-down with DISC1-siRNA-1 treatment, promotion of oligodendrocyte differentiation by DISC1 knockdown was inhibited (Fig. 6 G). The effect of DISC1-siRNA-1 on its target mRNA was not altered when either sox-siRNA or nkx-siRNA was co-transfected (Fig. 6 H). Similarly, DISC1-siRNA-1 co-transfection did not alter the effect of sox-siRNA or nkx-siRNA (Fig. 6 I, J). Therefore, these results suggest DISC1 negatively regulates oligodendrocyte differentiation by acting upstream of Sox10 and/or Nkx2.2 to suppress their expression.

## Discussion

The major findings of this study are as follows. First, we show that DISC1 is expressed in oligodendrocytes in the corpus callosum of postnatal mouse brain (Fig. 1). Second, DISC1 expression is decreased during *in vitro* differentiation of oligodendrocyte precursor cells to oligodendrocytes (Fig. 2 A). Third, DISC1 endogenously expressed in cells of an oligodendroglial lineage, negatively regulates differentiation of oligodendrocyte precursor cells to oligodendrocytes, as shown by promotion of oligodendrocyte differentiation by either DISC1 knockdown or overexpression of truncated DISC1 (Fig. 4, 5), while overexpression of full length DISC1 inhibits oligodendrocyte differentiation (Fig. 3). Finally, we have implicated Sox10 and/or Nkx2.2 in the DISC1 regulatory pathway of oligodendrocyte differentiation, with knockdown of endogenous DISC1 increasing, and DISC1 overexpression decreasing, expression of Sox10 and Nkx2.2. Additionally, promotion of oligodendrocyte differentiation by DISC1 knockdown was prevented by simultaneous knockdown of either Sox10 or Nkx2.2 (Fig. 6).

### DISC1 Expression in Oligodendrocytes

Our *in situ* hybridization analysis shows that DISC1 mRNA is expressed not only in oligodendrocytes of mouse brain but also in primary cultured rat oligodendrocytes and oligodendrocyte precursor cells (Fig. 1, 2). These results are consistent with previous report by Seshadri et al. showing colocalization of DISC1 with the oligodendrocyte marker in human brain and primary cultured rat cortical oligodendrocytes [37]. Furthermore, DISC1 expression in oligodendrocytes in the corpus callosum was higher in developing stage than in adulthood, similar to the developmental expression pattern of DISC1 in neurons [20], [46]. These results suggest that DISC1 has a developmental role in oligodendrocyte lineage cells as well as in neurons. Overexpressed DISC1 localized preferentially in cell bodies and processes of oligodendrocyte precursor cells and oligodendrocytes *in vitro*, with marginal nuclear localization (Fig. 2 B). The subcellular localization of DISC1 in oligodendrocyte precursor cells and oligodendrocytes is similar to that in neurons, suggesting the possibility that DISC1 has common functional roles between neurons and oligodendrocyte lineage cells.

### DISC1 Function in Oligodendrocytes

DISC1 has been shown to play an important role in immature neurons, regulating their differentiation, migration and proliferation [12], [17], [49]. Thus our findings, namely, decrease of DISC1 expression during the course of oligodendrocyte differentiation (Fig. 2 A), and higher DISC1 expression in oligodendrocytes in the mouse corpus callosum at P14 (Fig. 1 C), suggest DISC1 may also have a developmental role in immature oligodendroglial lineage cells as well. Supporting evidence is discussed below.

Overexpressed DISC1 disrupts not only induction of CNPase and MBP expression, but also transformation of oligodendrocytes to a complex morphology (Fig. 3), indicating a negative regulatory role of DISC1 in differentiation of oligodendroglial lineage cells *in vitro*. Conversely, both expression of CNPase and the number of matured oligodendrocytes, were increased when endogenously expressed DISC1 was knocked-down by siRNA, even if the cells were maintained in medium containing PDGF (Fig. 4). Although MBP mRNA levels were increased by DISC1 knockdown, the result did not reach statistical significance. This is likely due to both the transient nature of DISC1 knockdown by siRNA, compared to the more stable adenovirus overexpression system, and also that MBP expression increases at a later stage of differentiation than CNPase [50]. More robust increases of MBP expression may be observed at later time-points, with stronger and more continuous inhibition of DISC1. Truncated DISC1 is predicted to function in a dominant negative fashion, potentially by competing with full length DISC1 for interacting proteins. Thus promotion of oligodendrocyte differentiation by truncated DISC1 overexpression suggests a negative regulatory role for DISC1 in oligodendrocyte differentiation (Fig. 5). Further studies are needed to determine if DISC1 interacts with other proteins in oligodendrocyte lineage cells, as in neurons [51], [52]. Nevertheless, our results do indicate that decreased level of endogenous DISC1 promotes differentiation of oligodendrocyte precursor cells to oligodendrocytes.

To date, a functional role for endogenous DISC1 expressed in mammalian oligodendrocyte lineage cells has not been reported. A critical requirement for DISC1 in oligodendroglial development, by promoting specification of olig2-positive cells in the hindbrain and other brain regions of zebrafish, was reported by Wood *et al* [36]. Although this report also shows regulation of oligodendroglial development by DISC1, the low homology between zebrafish DISC1 and mammalian DISC1 (homologies between zebrafish and rat, mouse or human are 31, 32, 36% respectively), highlights the necessity of our study. Furthermore, it is not clear if neuronal or glial expressed DISC1 is responsible for oligodendroglial development. Katsel *et al*., showed that oligodendrocyte-associated gene/protein expression was changed in the forebrain of transgenic mice with forebrain restricted expression of mutant human DISC1 (ΔhDISC1) at embryonic, neonatal and adulthood stages [38]. The transgenic mice show neuron-specific overexpression of ΔhDISC1, therefore the observed alterations in oligodendrocyte-associated gene/protein expression are caused by mutant DISC1 expressed in neurons. However, the results of our study show that glial expressed DISC1 regulates oligodendrocyte differentiation. To further determine the role of glial expressed DISC1 on oligodendroglial development *in vivo,* studies using transgenic mice with glia-specific expression of mutant DISC1 are needed.

### Sox10, Nkx2.2 Regulation by DISC1

Each step of oligodendrocyte differentiation is under tight transcriptional control. Among many transcription factors acting at different stages, Sox10 and Nkx2.2 play a major role in the transition from oligodendrocyte precursor cells to pre-myelinating oligodendrocytes [48], [53]. Sox10 is a high-mobility group transcriptional regulator restricted in the central nervous system to myelin-forming oligodendrocytes. In Sox10-deficient mice, oligodendrocyte precursor cells develop but terminal differentiation is disrupted [54]. A critical role for the homeodomain-containing protein Nkx2.2 in the development of oligodendroglial lineage cells has also been reported. The number of MBP-positive and proteolipid protein (PLPDM-20)-positive oligodendrocytes are dramatically reduced in the brain of Nkx2.2 null mice [55]. Positive regulatory roles for Sox10 and Nkx2.2 in oligodendrocyte differentiation are consistent with our result that DISC1 regulates oligodendrocyte differentiation via upstream regulation of Sox10 and/or Nkx2.2 (Fig. 6). Negative regulation of Sox10 expression by DISC1 has previously been reported by Drerup *et al*., although they examined cranio-neural crest cells, which become glial precursors at later stages of development [56]. How DISC1 regulates these transcription factors is not yet known, but intracellular signaling pathways involving molecules such as Akt, cAMP, CREB and MAPK are likely candidates, as neuronal DISC1 regulates these pathways, and moreover, these signaling pathways have functional roles in differentiation of oligodendroglial lineage cells [57]–[62].

The pathophysiological role of Sox10 in SZ has been suggested by a report showing a correlative relationship between the DNA methylation status of the Sox10 gene and oligodendrocyte dysfunction in SZ [63]. In addition, a significant association in the genotype and allelic frequency of a single-nucleotide polymorphism of the Sox10 gene, between schizophrenic patients and controls has been reported [64]. Nkx2.2 is known to form a transcriptional network with Pet1, a molecule involved in differentiation of serotonergic neurons [65]. It is well known that serotonergic neurons are both a relevant pathophysiological factor and therapeutic target in several psychiatric diseases, including SZ, bipolar disorder, major depression and autism. Therefore our finding that DISC1, a key psychiatric disease susceptibility gene, controls Sox10 and/or Nkx2.2 expression is intriguing.

### Pathophysiological Role of DISC1 Regulated Oligodendrocyte Differentiation

Overall, our findings suggest that DISC1 dysfunction may cause impaired differentiation of oligodendrocytes by affecting Sox10 and/or Nkx2.2 expression, and consequently contribute to the pathophysiology of psychiatric disorders. Improper myelination of neuronal axons, resulting from impaired oligodendrocyte differentiation, may lead to defective neuronal communication, a likely component in the mechanistic background of “structural disconnectivity”, suggested in the pathophysiology of psychiatric disorders [24], [66]. Therefore, it would be of interest to investigate if WM abnormalities are a feature of the Scottish DISC1 pedigree that harbors the disrupted DISC1 gene. Furthermore, given that SZ-like psychosis co-occurs frequently in demyelinating diseases [67], it is also warranted to investigate the role of DISC1 in pathophysiology of demyelinating diseases such as MS, leukodystrophies and velocardiofacial syndrome.
